# Steady state analysis of Boolean molecular network models via model reduction and computational algebra

**DOI:** 10.1186/1471-2105-15-221

**Published:** 2014-06-26

**Authors:** Alan Veliz-Cuba, Boris Aguilar, Franziska Hinkelmann, Reinhard Laubenbacher

**Affiliations:** 1 Department of Mathematics, University of Houston, 651 PGH Building, Houston TX, USA; 2 Department of Biochemistry and Cell Biology, Rice University, W100 George R. Brown Hall, Houston TX, USA; 3 Department of Computer Science, Virginia Tech, Blacksburg VA, USA; 4 TNG Technology Consulting GmbH, Unterföhring, Germany; 5 Center for Quantitative Medicine, University of Connecticut Health Center and Jackson Laboratory for Genomic Medicine, Farmington CT, USA

**Keywords:** Steady state computation, Boolean model, Discrete model

## Abstract

**Background:**

A key problem in the analysis of mathematical models of molecular networks is the determination of their steady states. The present paper addresses this problem for Boolean network models, an increasingly popular modeling paradigm for networks lacking detailed kinetic information. For small models, the problem can be solved by exhaustive enumeration of all state transitions. But for larger models this is not feasible, since the size of the phase space grows exponentially with the dimension of the network. The dimension of published models is growing to over 100, so that efficient methods for steady state determination are essential. Several methods have been proposed for large networks, some of them heuristic. While these methods represent a substantial improvement in scalability over exhaustive enumeration, the problem for large networks is still unsolved in general.

**Results:**

This paper presents an algorithm that consists of two main parts. The first is a graph theoretic reduction of the wiring diagram of the network, while preserving all information about steady states. The second part formulates the determination of all steady states of a Boolean network as a problem of finding all solutions to a system of polynomial equations over the finite number system with two elements. This problem can be solved with existing computer algebra software. This algorithm compares favorably with several existing algorithms for steady state determination. One advantage is that it is not heuristic or reliant on sampling, but rather determines algorithmically and exactly all steady states of a Boolean network. The code for the algorithm, as well as the test suite of benchmark networks, is available upon request from the corresponding author.

**Conclusions:**

The algorithm presented in this paper reliably determines all steady states of sparse Boolean networks with up to 1000 nodes. The algorithm is effective at analyzing virtually all published models even those of moderate connectivity. The problem for large Boolean networks with high average connectivity remains an open problem.

## Background

Boolean network (BN) models are widely used in molecular and systems biology to capture coarse-grained dynamics of a variety of regulatory networks, with a particular focus on features such as steady state behavior [1-22]. One advantage of discrete models of this type is that, for small models, the entire dynamics can be explored by exhaustive enumeration of all state transitions. Since the size of the state space of a Boolean model with *n* nodes is 2^*n*^, this approach becomes unfeasible for larger models, those with more than approximately 30 variables, depending on the computational resources available. Also, for larger models, finding steady states (fixed points in this manuscript) through sampling is not effective anymore either, since even large attractors can be missed entirely by this approach. On the theoretical side, it has been shown that the problem of finding, or even counting, steady states of Boolean networks is NP-hard [23,24], so that any algorithm for this problem will eventually encounter serious limitations. Since the size of published models has increased in recent years, including models with 100 or more nodes [15,17,21,22], it is important to develop more efficient methods that find all steady states of a Boolean model.

Several methods have been proposed in the literature for dealing with this problem, including exact as well as heuristic methods. We provide a brief review of the different types here. For this purpose, we represent a Boolean network as follows. Let *K*={0,1}, and assume that the network has *n* nodes *x*_1_,…,*x*_*n*_. Each node *x*_*i*_ has associated to it a Boolean function *f*_*i*_:*K*^*n*^→*K*. Thus, we can represent the Boolean network as a function

$$f=(\;f_{1},\ldots ,f_{n}):K^{n}\to K^{n}.$$

One can represent the variable dependencies through the *dependency graph* of the network, whose nodes are the variables *x*_1_,…,*x*_*n*_. There is an edge *x*_*i*_→*x*_*j*_ if *x*_*i*_ appears in the function *f*_*j*_, that is, the state of *x*_*j*_ depends on the state of *x*_*i*_. The problem of finding steady states is then formulated as finding all states *x*∈*K*^*n*^ such that *f*(*x*)=*x*.

One approach to the problem is model reduction. Some existing *reduction methods* use a “steady-state approximation” [25-28] to reduce the number of variables. Intuitively, if a function depends on a variable, e.g., *f*_*i*_=*f*_*i*_(*x*_*j*_,*x*_*k*_,*x*_*l*_), then we can remove variable *x*_*j*_ from the network by replacing *f*_*i*_(*x*_*j*_,*x*_*k*_,*x*_*l*_) with the new function *f*_*i*_(*f*_*j*_(*x*_1_,…,*x*_*n*_),*x*_*k*_,*x*_*l*_). By repeating this process, one obtains a reduced network that in practice is much smaller than the original network. The stopping criteria for reduction methods is that variables can be removed only if the steady state information is preserved. The steady states of the reduced network are in algorithmic one-to-one correspondence with the steady states of the original network. More precisely, the reduction algorithm decomposes a large system into a smaller system and a set of equations in triangular form, so that when the steady states of the reduced system are found, the steady states of the original systems can be found simply by backwards substitution. That is, the existence of the one-to-one correspondence is not just theoretical.

Another method uses the fact that one can represent a Boolean function as a polynomial function in the variables *x*_1_,…,*x*_*n*_, with coefficients in the finite number system *K*={0,1} (with integer addition and multiplication modulo 2). The problem of finding the steady states of a Boolean network in *n* variables, as above, can then be reformulated as the problem of finding the solutions to a system of polynomial equations *p*_*i*_:=*f*_*i*_(*x*_1_,…,*x*_*n*_)−*x*_*i*_=0;*i*=1,…,*n*[29-31]. Then, the roots of the system of polynomial equations is encoded by the set {*p*_1_,…,*p*_*n*_}. Using tools from computational algebra it is possible to find another set that has the same roots (a Gröbner basis), such that it is possible to do a generalized version of Gaussian elimination. These computations can be done using several different software packages developed for this purpose.

A graph-theoretic method, *Minimal Feedback Vertex Sets*, consists of finding a set of vertices in the dependency graph of the network that “generate” all steady states. More precisely, one finds a set *S*⊂{1,…,*n*} such that knowing the coordinates *x*_*i*_, for all *i*∈*S*, of a steady state completely and algorithmically determines the other coordinates of the steady state. It turns out that so-called feedback vertex sets have this property. In practice, by finding a minimal feedback vertex set, one reduces the problem from checking 2^*n*^ states to the problem of checking 2^|*S*|^ states, where |*S*| is typically much smaller than *n*[23]. A feedback vertex set can be found by removing vertices from the graph until the graph has no directed cycles. A minimal feedback vertex set can be found by finding the smallest number of vertices that we need to remove from the graph so that it does not have directed cycles.

SAT methods have also been used for the purpose of finding steady states of Boolean networks, which are used to determine whether a Boolean expression in several variables has a variable assignment that makes the expression true; see [32-35]. In essence, the system of Boolean equations, *f*_*i*_=*x*_*i*_, is rewritten as a single equation *G*(*x*)=1, and then the problem of finding the steady states becomes the problem of finding when the equation *G*(*x*)=1 is satisfied. For example, Melkman, Tamura, and Akutsu [33,35] used SAT algorithms to find steady states of AND/OR Boolean networks, i.e., Boolean networks in which the *f*_*i*_ contain only the AND and OR operators, with a time complexity of *O*(1.587^*n*^) (where *n* is the number of nodes). Dubrova and Teslenko [34] also developed a SAT-based algorithm to find all attractors of a Boolean network with very good performance characteristics. The methodology was tested on Boolean networks with sizes ranging from 12 to 52. It was also tested using random networks with up to 7000 nodes and average in-degree less than 2. For a fixed in-degree of 2 the maximum size networks tested have 2000 nodes.

Integer programming-based method have also been used to find the steady states of Boolean networks, Tamura, Hayashida, and Akutsu [36]. In essence, the system of Boolean equations is rewritten as a set of inequalities *Ax*≤*b*,*x*≥0 and the goal is to maximize a linear function of the form *c*^*T*^*x*.

*Strategic Sampling*, (Zhang, Hayashida, Akutsu, Ching, and Ng, [37]) is a recursive search approach to identify all steady states of a random Boolean network with maximum in-degree 2, with an average time complexity of *O*(1.19^*n*^) (where *n* is the number of nodes). The idea is that the equations are solved recursively: First one considers the solutions of the equation *f*_1_=*x*_1_. Since the *f*_*i*_’s depend on few variables in practice, one only has to keep track of the variables that appear in *f*_1_. Then, one finds the solutions of *f*_2_=*x*_2_ that are compatible with the solutions previously found. The process continues until one finds solutions of all equations. In the worst case, however, algorithm complexity can be *O*(*n*2^*n*^) [31].

Finally, the problem of finding attractors has also been studied by using Binary Decision Diagrams (BDD) [38-41]. The idea is to represent the Boolean functions as a directed graph that efficiently encodes the functions by allowing fast evaluation. Then, by combining the BDD representation of all the Boolean functions, the problem of finding steady states becomes a search problem in the larger BDD. Many of these methods were tested on some biologically relevant networks with fewer than 100 nodes.

In this paper, we present a new method for computing steady states of a Boolean network, combining a graph theoretic reduction/transformation method with an approach using computational algebra. We show that the method performs favorably on some types of networks in comparison with other methods on a collection of benchmark networks, consisting of both published models and random networks with certain properties, namely Kauffman networks and networks whose in-degree distribution satisfies a power law.

## Methods

**Figure 1:**
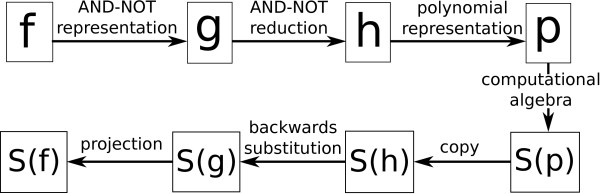
**Flow chart of steady state computation.** Main steps in our method highlighting the intermediate systems. *S* denotes the set of steady states of a given network; *f* is an arbitrary Boolean network, *g* is an AND-NOT network (in possibly more variables), *h* is a reduced AND-NOT network, *p* is the polynomial representation of *h*.

The method we propose for steady state analysis is a combination of network reduction/transformation and computational algebra (see Figure 1). The reduction technique we use is based on results in [42,43]. In [42] it was shown that any Boolean network can be “transformed” into an AND-NOT network, namely a network whose Boolean functions are all of the form *y*_1_∧*y*_2_∧…, where *y*_*i*_∈{*x*_*i*_,¬*x*_*i*_}. The AND-NOT network has the property that its steady states are in one-to-one correspondence with the steady states of the original network. Furthermore, the one-to-one correspondence between steady states is algorithmic. In [43], the authors proposed a method to reduce an AND-NOT network to another, smaller AND-NOT network in polynomial time, in such a way that the steady states of the original and the reduced network are in one-to-one correspondence, in a constructive way. This reduction algorithm looks for motifs (e.g. feed-forward loops) in the wiring diagram and removes nodes in such motifs; the reduction stops when there are no more motifs to be reduced (attempting to do further reductions would destroy the 1-1 correspondence of steady states). Once the reduced network is constructed, one can compute its steady states by converting the Boolean functions into polynomial functions and then solving a system of polynomial equations, as explained above. The computational algebra technique is based on [29,30]. The idea is that by computing a Gröbner basis (a special set of polynomials with the same roots as the original equations), it is possible to find the roots of the system of polynomial equations using a generalized version of Gaussian elimination.

The correspondence between Boolean and polynomial functions is accomplished via the “dictionary” *x*∧*y*⇔*x*·*y*,*x*∨*y*⇔*x*+*y*+*xy*,¬*x*⇔*x*+1. The correspondence is unique if we limit the degree with which each variable appears in the polynomial function to 1, since any function *K*^*n*^→*K* can be represented uniquely as a polynomial function that is square-free, that is, in which every variable appears with exponent 1.

The algorithm is summarized in the following pseudocode and a more detailed description follows. The source code can be found at github.com/PlantSimLab/ ADAM. 

The input of our algorithm is an *n*−dimensional Boolean network *f*=(*f*_1_,…,*f*_*n*_). In Step 1, we use the formulas from [42] to compute an AND-NOT network *g*=(*g*_1_,…,*g*_*m*_), with *m*≥*n*, which has the same number of steady states as *f*. The idea is to introduce variables to rewrite the Boolean operations using only the operators AND and NOT; for example, *f*_1_=¬*x*_2_∧(*x*_3_∨*x*_4_) can be written as *f*_1_=¬*x*_2_∧¬*x*_5_, where *f*_5_=¬*x*_3_∧¬*x*_4_. Furthermore, the steady states of *f* are given by projecting the steady states of *g* to their first *n* coordinates. In Step 2, we simply consider the wiring diagram of *g*, which is a signed directed graph that encodes which variable depends on which others and whether the interactions are activating or inhibiting. In Step 3, we use the algorithm from [43] to reduce the wiring diagram of *g* to another signed directed graph, *W*. Then, in Step 4, we construct the AND-NOT network that has *W* as its wiring diagram, *h*=(*h*_1_,…,*h*_*l*_); the steady states of *g* can be computed from the steady states of *h* by backtracking [43]. In Step 5, we compute the polynomial representation of *h*. This is done by replacing ¬*x*_*i*_ with 1+*x*_*i*_, and *x*_*i*_∧*x*_*j*_ with *x*_*i*_*x*_*j*_, as explained earlier. In Step 6 we solve the system of polynomial equations *h*_*i*_=*x*_*i*_, *i*=1,…,*l*; this is done using tools from computational algebra as done in [29,30]. The solutions of the system, *L*^″^={*s*1″,…,*sr*″}, will also be solutions of *h*. In Step 7, we use backtracking to compute the steady states of *g*, *L*^′^={*s*1′,…,*sr*′}. And finally, in Step 8, we project each *sj*′ to its first *n* coordinates and obtain the steady states of *f* (See Additional file 1 for an example and Additional files 2 and 3 for the code version used for this publication).

## Results and discussion

We first tested the software implementation of our algorithm on 1,000,000 Boolean networks with 50 nodes each, for which we also computed all steady states by a custom-made algorithm based on minimal feedback vertex sets. For each graph we found the minimal number of vertices that had to be removed so that the graph had no directed cycles; call this set *S*. Then, for each element in {0,1}^|*S*|^, the values of the other variables are completely determined. This gave us 2^|*S*|^ candidates for steady states which we then checked by exhaustive search. In all cases our algorithm computed correctly all steady states. We are therefore confident that our implementation is error-free. This extends to the relevant functionalities of other software packages we used for intermediate computations (Macaulay2 [44], Boost Library [45], BoolStuff Library [46]).

Then we used over 100,000 Boolean networks to benchmark our method against others. The methods we used for comparison were those with published benchmarks or those for which the code was readily available. As we will see later, for Kauffman networks with *K*=2, the timing of our method grows linearly with the number of nodes; thus, it was not necessary to include in our benchmarks methods that were reported to grow exponentially for such networks (e.g. [34,37]). We selected three methods with good computational efficiency for *K*=2: Zanudo and Albert [26]; Devloo, Hansen, and Labbé [32]; and Tamura, Hayashida, and Akutsu [36]. For the most recent algorithm, Zañudo and Albert [26] use a method that identifies motifs (subsets of nodes) that stabilize in one or a small number of states. The steady states from these motifs are used to reduce the network to find the attractors. It is important to mention that this method can find not only the steady states of Boolean networks, but also information about all the attractors of the network, which our method is not currently designed to do.

We used random biologically meaningful Boolean networks [47-49] and published networks [13-22] (the Boolean representation of these models was obtained from *The Cell Collective*[50]). The results for Zañudo and Albert and our algorithm were generated by us and the other results are reported from published benchmarks [32,36]. The computations for our algorithm and that of [26] were done on a 3.4 GHz Linux machine. The computations for Tamura’s and Devloo’s algorithms were done on a Linux system with 3GHz and a Sun SPARC Ultra 10 machine, respectively, as reported in [32,36]. Considering that the different computers described above have processors with similar speed and that the computations were done in a single processor, the use of results from different machines will not affect the main conclusions of our comparison. Moreover, some methods did not have reported results for certain network sizes; in that case, we computed an approximate timing using interpolation/extrapolation of the reported values; we linear and exponential fits for the timings that grew linearly and exponentially, respectively.

First, we compare the performance of different methods on Kauffman networks with connectivity *K*=2 and *K*=3. For our and the Zañudo algorithm, each reported number is the average or standard deviation of 1000 Boolean networks. In Table 1 we report the timings for Kauffman networks with *K*=2. We can see that the algorithm in [36] performs best, followed by our algorithm. Note that all timings grow linearly with the number of nodes. As mentioned in [36], the good results with Tamura’s algorithm may be due to the fact that the authors optimized the computations for Boolean functions that have 2 inputs. The results for Kauffman networks with *K*=3 in Table 2, however, show that our method performs better by an order of magnitude. These results show that, while our algorithm is not optimized for very low in-degree networks, it is more scalable for networks with higher connectivity.

**Table 1:** **Timing in seconds for Kauffman networks with** ***K=2***

	**Zañudo **[26]		**Devloo **[32]		**Tamura **[33]		**Our method**	
***n***	**mean**	**stdev.**	**mean**	**stdev.**	**mean**	**stdev.**	**mean**	**stdev.**
2000	7.341	3.192	107.1 ^∗^	83.49 ^∗^	**0.022**	NR	0.490	0.023
4000	12.084	3.636	223.0 ^∗^	173.9 ^∗^	**0.035**	NR	1.123	0.049
6000	31.174	340.213	338.9 ^∗^	264.3 ^∗^	**0.047**	NR	2.172	0.114
8000	28.091	11.572	454.8 ^∗^	354.8 ^∗^	**0.069**	NR	3.642	0.212
10000	38.394	13.301	570.8 ^∗^	445.2 ^∗^	**0.072**	NR	5.218	0.235

The best results are in bold. *=interpolated/extrapolated from reported results. NR=not reported.

**Table 2:** **Timing in seconds for Kauffman networks with** ***K=3***

	**Zañudo **[26]		**Devloo **[32]		**Tamura **[33]		**Our method**	
***n***	**mean**	**stdev.**	**mean**	**stdev.**	**mean**	**stdev.**	**mean**	**stdev.**
20	1.024	0.403	0.110	0.090	**0.011**	NR	0.273	0.040
40	DF	DF	0.340	0.270	**0.296**	NR	0.300	0.126
60	DF	DF	2.251 ^∗^	2.120 ^∗^	2.414	NR	**0.415**	0.552
80	DF	DF	10.05 ^∗^	10.84 ^∗^	17.07	NR	**1.143**	8.414
100	DF	DF	60.10	59.10	94.08	NR	**2.878**	16.74
120	DF	DF	200.5 ^∗^	283.6 ^∗^	714.4 ^∗^	NR	**9.278**	51.79

The best results are in bold. *=interpolated/extrapolated from reported results. DF=did not finish in a day. NR=not reported.

Not all molecular networks have properties similar to Kauffman networks, but can exhibit power law properties for their degree distribution. Thus, we supplemented the results from Tables 1 and 2 with benchmark networks whose connectivity follows a power law distribution [51]. We considered power-law networks with average connectivity 〈*k*〉=2 and 〈*k*〉=3. That is, the average number of edges is the same, but the connectivity distribution is more biologically realistic. There were no bechmarks for these types of networks for Tamura’s and Devloo’s algorithms, so we only report Zañudo’s and our algorithm. In Table 3, we see that our algorithm can handle networks with 〈*k*〉=2 with up to 1000 nodes in under 7 seconds on average. It is important to mention that these timings differ considerably from the timings for *K*=2 (Table 1). Table 4 shows the results for networks with connectivity 〈*k*〉=3. Not surprisingly, increasing the average connectivity has a dramatic effect on the size of networks that can be studied; for example, the network sizes that can be dealt with in under 7 seconds decreases from 1000 to about 140 when we increase 〈*k*〉 from 2 to 3. Further increasing the average connectivity will have a much more dramatic effect.

**Table 3:** **Timing in seconds for power-law networks with average connectivity** ***〈k〉=2***

	**Zañudo **[26]		**Our method**	
***n***	**mean**	**stdev.**	**mean**	**stdev.**
25	1.264	1.778	0.254	0.011
50	2.488	3.807	0.257	0.018
100	5.255	9.172	0.260	0.022
250	DF	DF	0.271	0.046
500	DF	DF	0.358	1.429
1000	DF	DF	6.798	65.39

DF = did not finish in a day.

**Table 4:** **Timing in seconds for power-law networks with average connectivity** ***〈k〉=3***

	**Zañudo **[26]		**Our method**	
***n***	**mean**	**stdev.**	**mean**	**stdev.**
20	3.828	5.133	0.251	0.029
40	DF	DF	0.259	0.055
60	DF	DF	0.288	0.222
80	DF	DF	0.543	4.724
100	DF	DF	1.331	7.752
120	DF	DF	3.033	25.94
140	DF	DF	7.185	57.23

DF = did not finish in a day.

Finally, our results on published networks are shown in Table 5, sorted by average connectivity. Since all models have external parameters corresponding to environmental conditions (i.e. we have one BN for each parameter set), we sampled the parameter space and computed the average timing of each algorithm. The numbers we report are the averages of 10000 simulations for each model. As expected, for all networks with small average connectivity (less than 3) our algorithm performed very well and finished in less than half a second, consistent with the timings from Tables 3 and 4. Four models have average connectivity greater than 3 and our algorithm performed very well on three of them. However, for the largest network (225 nodes and 〈*k*〉=5.16), there were parameter sets (51% of the sampled parameters) which could not be analyzed.

**Table 5:** Timimg in seconds for published models

			**Zañudo **[26]		**Our method**	
**Ref.**	***n***	***〈k〉***	**mean**	**stdev.**	**mean**	**stdev.**
[13]	62	1.62	1.678	0.729	0.231	0.010
[14]	94	1.65	1.300	0.074	0.234	0.012
[15]	302	1.71	4.698	0.116	0.236	0.011
[16]	60	2.10	4636.245	89.311	0.239	0.013
[17]	120	2.45	2023.954	18448.754	0.312	0.141
[18]	54	2.59	6878.594	22059.317	0.256	0.030
[19]	54	3.62	3.789	3.903	0.492	0.247
[20]	76	4.01	DF	DF	0.242	0.013
[21]	130	5.00	DF	DF	23.19	98.42
[22]	225	5.16	DF	DF	4186*	12284

DF=did not finish in a day. *=49% of simulations reported, 51% of simulations were stopped because they did not finish in a day or had a large memory consumption.

The computational complexity of our algorithm depends on the type of networks used as well as the connectivity. The algorithm seems to run in polynomial time for Kauffman networks with *K*=2 (Table 1), but slower for power-law networks with the same connectivity (Table 3). For other types of networks the complexity is much harder to infer, but Table 2 suggests that the complexity is exponential. Also, the complexity of the mathematical tools we use is not well understood in the context of Boolean models. For example, the algebraic step of our algorithm can be doubly exponential, but it has been shown to work much faster in practice and, as our work shows, it runs much faster for sparse Boolean models.

## Conclusions

The capability to analyze the attractors of discrete dynamic models of biological networks is a key technology in any systems biology toolkit that incorporates this popular type of model. This capability needs to include steady state analysis as well as the determination of periodic points of larger periods. And it needs to apply to models that allow an arbitrary (finite) number of states for its variables, such as logical models. In this paper, we have focused on Boolean networks as the model type most commonly used currently. And we have focused only on steady state analysis, at the exclusion of periodic limit cycles. As is the case in many situations, algorithms available for this purpose, some of which we used here for comparison, perform well on some types of models and not so well on others. For instance, for Kaufmann networks with connectivity 2, the method in [36] outperforms all other methods, including ours. The method in [26] is generally slower than our method in computing steady states, but has the added capability that it also finds limit cycles of larger lengths, which our method is not currently equipped to do.

We have used three types of networks for benchmarking: Kauffman networks, power law networks, and published networks. Kauffman networks are commonly used for this purpose, but they don’t capture all properties of molecular networks, which include a power law distribution of node connectivities. Our analysis of published networks shows that some of them have high average connectivity, not generally considered in theoretical studies. These pose serious challenges to computational methods, as we demonstrate. As more large published networks become available, they will represent the most important suite of benchmark models to be used, in our opinion.

We believe that this study also holds another important lesson. Our method is a combination of two methods, neither one of which performs particularly well when applied on its own (see Additional file 1). In combination, however, they are quite powerful: model reduction plus polynomial algebra. This might point towards a general strategy for other algorithms of this type. Nonetheless, as our calculations show, the challenge of finding steady states is far from solved in general, even for existing published models. Thus, much work remains to be done.

## Competing interests

The authors declare that they have no competing interests.

## Authors’ contributions

AV-C designed and applied the graph reduction methods, and combined them with the computer algebra algorithm. He also generated the suite of benchmark networks used in the study. BA implemented the graph reduction methods. He also surveyed the literature for other available methods and carried out and collected performance data for the other methods used in the study for comparison. FH carried out Gröbner basis calculations for a subset of the benchmark networks. RL conceived, planned, and directed the project. All authors contributed to the writing of the manuscript. All authors read and approved the final manuscript.

## Supplementary Material

Additional file 1 Example and individual performance of network reduction and computational algebra. Click here for file

Additional file 2 Instructions for usage. Click here for file

Additional file 3 Source code. Click here for file
