# Supplementary material for: Steady state analysis of Boolean molecular network models via model reduction and computational algebra
Source: BMC Bioinformatics. 2014 Jun 26;15:221. doi: 10.1186/1471-2105-15-221 (PMC4230806; doi:10.1186/1471-2105-15-221)
Supplement: Additional file 1 — Example and individual performance of network reduction and computational algebra. [file 1471-2105-15-221-S1.pdf]

# STEADY STATE ANALYSIS OF BOOLEAN MOLECULAR NETWORK MODELS VIA MODEL REDUCTION AND COMPUTATIONAL ALGEBRA.

ALAN VELIZ-CUBA, BORIS AGUILAR, FRANZISKA HINKELMANN, REINHARD LAUBENBACHER

## Example

Here we show an example of how our algorithm works. We use the Th-cell differentiation model from [1]:

$$
\begin{array}{lllllll}
x_1 & = & GATA3 & , & f_1 & = & (x_1 \vee x_{21}) \wedge \neg x_{22};\\
x_2 & = & IFN-\beta & , & f_2 & = & 0;\\
x_3 & = & IFN-\beta R & , & f_3 & = & x_2;\\
x_4 & = & IFN-\gamma & , & f_4 & = & (x_{14} \vee x_{16} \vee x_{20} \vee x_{22}) \wedge \neg x_{19};\\
x_5 & = & IFN-\gamma R & , & f_5 & = & x_4;\\
x_6 & = & IL-10 & , & f_6 & = & x_1;\\
x_7 & = & IL-10R & , & f_7 & = & x_6;\\
x_8 & = & IL-12 & , & f_8 & = & 0;\\
x_9 & = & IL-12R & , & f_9 & = & x_8 \wedge \neg x_{21};\\
x_{10} & = & IL-18 & , & f_{10} & = & 0;\\
x_{11} & = & IL-18R & , & f_{11} & = & x_{10} \wedge \neg x_{21};\\
x_{12} & = & IL-4 & , & f_{12} & = & x_1 \wedge \neg x_{18};\\
x_{13} & = & IL-4R & , & f_{13} & = & x_{12} \wedge \neg x_{17};\\
x_{14} & = & IRAK & , & f_{14} & = & x_{11};\\
x_{15} & = & JAK1 & , & f_{15} & = & x_5 \wedge \neg x_{17};\\
x_{16} & = & NFAT & , & f_{16} & = & x_{23};\\
x_{17} & = & SOCS1 & , & f_{17} & = & x_{18} \vee x_{22};\\
x_{18} & = & STAT1 & , & f_{18} & = & x_3 \vee x_{15};\\
x_{19} & = & STAT3 & , & f_{19} & = & x_7;\\
x_{20} & = & STAT4 & , & f_{20} & = & x_9 \wedge \neg x_1;\\
x_{21} & = & STAT6 & , & f_{21} & = & x_{13};\\
x_{22} & = & T-bet & , & f_{22} & = & (x_{18} \vee x_{22}) \wedge \neg x_1;\\
x_{23} & = & TCR & , & f_{23} & = & 0.
\end{array}
$$

The wiring diagram is shown in Figure 1. The AND-NOT representation[1] of this Boolean network is shown in Figure 2 and the reduced AND-NOT network is shown in Figure 3.

The polynomial representation of the reduced AND-NOT network is

$$f_1 = x_1 x_{22} + x_1,$$

$$f_{22} = x_1 x_{22} + x_{22}.$$

To compute the steady states we need to solve

$$x_1 = x_1 x_{22} + x_1,$$

$$x_{22} = x_1 x_{22} + x_{22},$$

[1]The AND-NOT representation is not unique and this particular representation was selected by hand for an easier comparison between the original Boolean network and its AND-NOT representation.

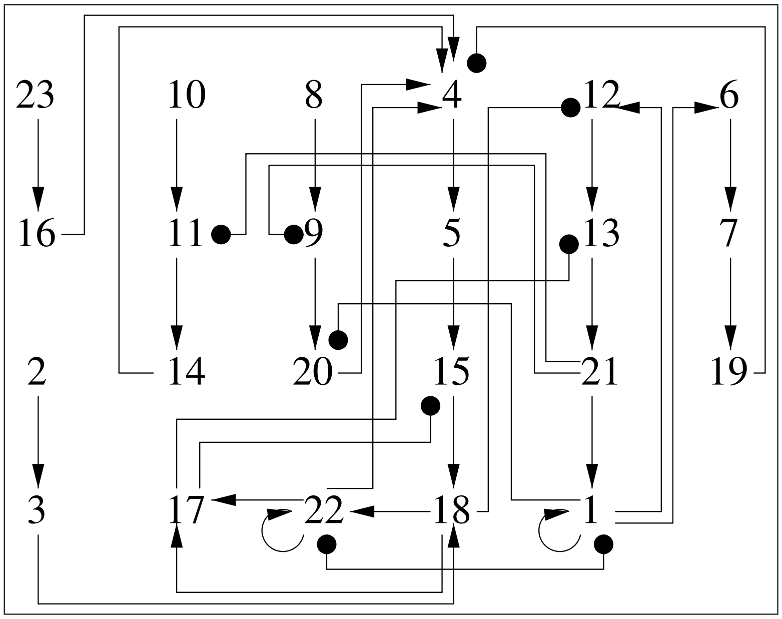


Figure 1. Wiring diagram of original Boolean network.

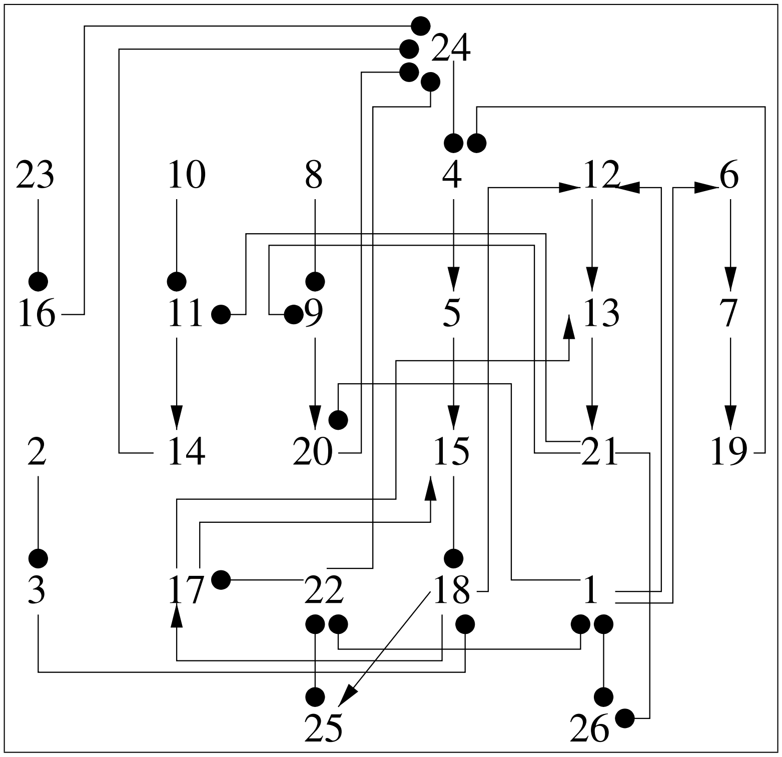


Figure 2. Wiring diagram of the AND-NOT network representation.

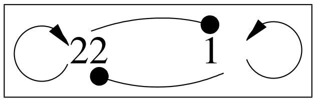


Figure 3. Wiring diagram of the reduced AND-NOT network.

that is,

$$0 = x_1 x_{22},$$

which is clearly satisfied for $(x_1, x_{22}) = (0,0), (0,1), (1,0)$. Now that we have the steady states of the reduced network, we can use backwards substitution to obtain the steady states of the larger AND-NOT network and then project on the variables of the original Boolean network; we obtain $x =$ 0000000000000000000000, 0001100000000001000010, 10000110000110000010100 (parenthesis were omitted). The first steady state corresponds to the "naive" Th-cell and the other two steady states correspond to the so-called Th-1 and Th-2 cells in charge of proinflammatory and anti-inflammatory responses in the immune system, respectively [1].

To use our code, the Boolean network has to be written in the form

```
(x1 | x21 ) & !x22
0
x2
(x14 | x16 | x20 | x22 ) & !x19
x4
x1
x6
0
x8 & !x21
0
x10 & !x21
x1 & !x18
x12 & !x17
x11
x5 & !x17
x23
x18 | x22
x3 | x15
x7
x9 & !x1
x13
(x18 | x22 ) & !x1
0
```

and one can simply run

```
./BNReduction.sh input_file
```

where input_file is the file shown above (all steps and "piping" are done automatically). The steady states will be printed in a file named input_file.fp:

```
user@computer:~$ ./BNReduction.sh input_file; cat input_file.fp
0000000000000000000000
0001100000000001000010
10000110000110000010100
```

## Comments about performance of reduction and polynomial algebra

In the manuscript we combined two main methods: (AND-NOT) network reduction and computational algebra [2, 3, 4, 5]. Also, in order to use the reduction of AND-NOT networks, we had to use the intermediate step of transforming a Boolean network into an AND-NOT network [6].

The reduction method by itself does not provide any means for computing steady states, it only guarantees that the reduced AND-NOT network has fewer variables. The reduction algorithm has to ensure that the number of steady states is preserved, so in many cases even the reduced AND-NOT network is too large to analyze by exhaustive search. For example, for Kauffman networks with $k = 3$ and $n = 100$, about 40% of the reduced AND-NOT networks had more than 50 variables. Using polynomial algebra allows to fill the gap and complete steady state computation (Table 2 in manuscript).

The polynomial algebra approach does provide a way to compute steady states, but it has trouble handling a large number of variables. For example, even with 500 variables and Kauffman networks with $k = 2$, the timings were 80s on average; for larger networks the timings are worse. However, by using the reduction method as a preprocessing step, the polynomial algebra approach can handle thousands of variables (Table 1 in manuscript).

## References

[1] Luis Mendoza and Ioannis Xenarios. A method for the generation of standardized qualitative dynamical systems of regulatory networks. *Theoretical Biology and Medical Modelling*, 3(1):13, 2006.

[2] A. Veliz-Cuba, R. Laubenbacher, and B. Aguilar. Dimension reduction of large AND-NOT network models. *arxiv.org/abs/1311.6868*, 2013.

[3] A. Veliz-Cuba, A. Jarrah, and R. Laubenbacher. Polynomial algebra of discrete models in systems biology. *Bioinformatics*, 26(13):1637–1643, 2010.

[4] Franziska Hinkelmann, Madison Brandon, Bonny Guang, Rustin McNeill, Grigoriy Blekherman, Alan V. Cuba, and Reinhard Laubenbacher. ADAM: Analysis of discrete models of biological systems using computer algebra. *BMC Bioinformatics*, 12(1):295+, 2011.

[5] D. Grayson and M. Stillman. Macaulay 2, a software system for research in algebraic geometry. Available at http://www.math.uiuc.edu/Macaulay2/.

[6] A. Veliz-Cuba, K. Buschur, R. Hamershock, A. Kniss, E. Wolff, and R. Laubenbacher. AND-NOT logic framework for steady state analysis of Boolean network models. *Applied Mathematics and Information Sciences*, 4(7):1263–1274, 2013.
